# Supplementary material for: Fecal Immunochemical Tests Detect Screening Participants with Multiple Advanced Adenomas Better than T1 Colorectal Cancers
Source: Cancers (Basel). 2021 Feb 5;13(4):644. doi: 10.3390/cancers13040644 (PMC7914536; doi:10.3390/cancers13040644)
Supplement: Supplementary file 1 [file cancers-13-00644-s001.pdf]

# Supplementary Materials: Fecal Immunochemical Tests Detect Screening Participants with Multiple Advanced Adenomas Better than T1 Colorectal Cancers: FITs by CRC Stage and AA Characteristics

**Anton Gies, Tobias Niedermaier, Laura Fiona Gruner, Thomas Heisser, Petra Schrotz-King and Hermann Brenner**

**Table S1.** T staging definitions for colorectal cancer according to the AJCC Cancer Staging Manual, Seventh Edition (2010) and resulting TNM classifications.

| T Stage | Definition/Description | | |
|---|---|---|---|
| T1 | Tumor growth into (but not through) submucosa | | |
| T2 | Tumor growth into (but not through) muscularis propria | | |
| T3 | Tumor growth through muscularis propria into subserosa, or into non-peritonealised pericolic/perirectal tissues | | |
| T4 | Penetration of the visceral peritoneal layer or penetration or adhesion to adjacent organs | | |
| | **TNM Classification Manual, 7th Edition:** | | |
| **AJCC/UICC** | **TNM** | **Dukes** | **Comment** |
| I | T1N0M0 | A | |
| | T2N0M0 | A | |
| II | T3N0M0 | B | |
| | T4aN0M0 | B | |
| | T4bN0M0 | B | |
| III | T1-2N1M0 | C | |
| | T1N2aM0 | C | |
| | T3N1M0 | C | |
| | T2N2aM0 | C | |
| | T3N2aM0 | C | |
| | T1N2bM0 | C | |
| | T2N1cM0 | C | |
| | T2N2bM0 | C | |
| | T4aN1aM0 | C | |
| | T4aN2aM0 | C | |
| | T3N2bM0 | C | |
| | T4aN2bM0 | C | |
| | T4aN1bM0 | C | |
| | T4aN1cM0 | C | |
| | T4bN1aM0 | C | |
| | T4bN1bM0 | C | |
| | T4bN2aM0 | C | |
| | T4bN2bM0 | C | |
| IV | M1a | D | Metastasized to one organ or site, any T, any N |
| | M1b | D | Metastasized to more than one organ or site, any T, any N |

AJCC, American Joint Committee on Cancer; UICC, Union for International Cancer Control.

**Table S2.** Sensitivity (% (95%CI)) for AA by size at cutoffs yielding 93% specificity.

| Variable | *n* | IDK Hb ELISA | QuantOn Hem | immoCARE-C | RIDASCREEN Hb | CAREprime | SENTiFIT-FOB Gold | Eurolyser FOB test | OC-Sensor |
|---|---|---|---|---|---|---|---|---|---|
| **Overall** | 200 | 31.5 (25.1–38.4) | 28.0 (21.9–34.8) | 29.7 (23.4–36.5)* | 31.0 (24.7–37.9) | 29.5 (23.3–36.3) | 28.5 (22.4–35.3) | 31.0 (24.7–37.9) | 26.5 (20.5–33.2) |
| ***by size***** | | | | | | | | | |
| <5 mm | 21 | 9.5 (1.2–30.4) | 4.8 (0.1–23.8) | 4.8 (0.1–23.8) | 4.8 (0.1–23.8) | 4.8 (0.1–23.8) | 4.8 (0.1–23.8) | 4.8 (0.1–23.8) | 4.8 (0.1–23.8) |
| ≥5–10 mm | 34 | 23.5 (10.8–41.2) | 17.7 (6.8–34.5) | 29.4 (15.1–47.5) | 23.5 (10.8–41.2) | 23.5 (10.8–41.2) | 26.5 (12.9–44.4) | 29.4 (15.1–47.5) | 20.6 (8.7–37.9) |
| ≥10–30 mm | 108 | 36.1 (27.1–45.9) | 36.1 (27.1–45.9) | 32.7 (24.0–42.5)* | 37.0 (27.9–46.9) | 34.3 (25.4–44.0) | 31.5 (22.9–41.1) | 35.2 (26.2–45.0) | 30.6 (22.1–40.2) |
| ≥30 mm | 12 | 41.7 (15.2–72.3) | 41.7 (15.2–72.3) | 41.7 (15.2–72.3) | 41.7 (15.2–72.3) | 41.7 (15.2–72.3) | 50.0 (21.1–78.9) | 33.3 (10.0–65.1) | 41.7 (15.2–72.3) |
| *p (trend)* | | **0.0094** | **0.0010** | **0.0240** | **0.0029** | **0.0056** | **0.0065** | **0.0349** | **0.0063** |

*Analysis based on one less AA case.; **25 of the 200 AA–cases were not included in this analysis, because grouping into the four size categories was not possible.

Abbreviations: AA, advanced adenoma; CI, confidence interval.
